# Supplementary material for: Regulation of lipid saturation without sensing membrane fluidity
Source: Nat Commun. 2020 Feb 6;11:756. doi: 10.1038/s41467-020-14528-1 (PMC7005026; doi:10.1038/s41467-020-14528-1)
Supplement: Supplementary file 1 — Supplementary Information [file 41467_2020_14528_MOESM1_ESM.pdf]

Supplementary Materials

for Ballweg *et al*.
Regulation of lipid saturation without sensing membrane fluidity

(contains Supplementary Methods, Supplementary Figures 1-7, and Supplementary Tables 1-5)

Correspondence and requests for materials should be addressed to Robert Ernst (email: robert.ernst@uks.eu)

Supplementary Methods

Reagents and antibodies.
All chemicals and reagents were of analytical or higher grade and obtained from Sigma Aldrich if not stated otherwise. The following antibodies were used: mouse anti-Myc (9E10), mouse anti-Pgk1 (Life Technologies), mouse anti-MBP (NEB), anti-mouse-HRP (Dianova), anti-mouse-IRDye 800CW (LI-COR). Atto488-PE was purchased from AttoTec GmbH. Abberior Star Red-Cholesterol is purchased from Abberior GmbH. It has a PEG linker between cholesterol moiety and the fluorescent tag.

Cultivation and genetic manipulation of *S. cerevisiae*
Plasmids (Table1) were used for the transformation of baker's yeast (Table 2). Overnight cultures were inoculated from single colonies and cultivated in SCD selection medium at 30°C until the stationary phase was reached. The UFA auxotroph *ΔSPT23ΔMGA2* strain was cultivated in the presence of 0.05% sodium linoleate. Main cultures were inoculated to an OD600 of 0.2 in rich medium (YPD) and cultivated to the mid-exponential phase (OD600 ≈ 1.0). If indicated, the YPD was supplemented with sodium linoleate.
A *CEN*-based plasmid expressing 3xmyc-tagged *MGA2* under the control of the *MGA2* promotor for near-endogenous levels was used as described previously [1]. Mutagenesis of *MGA2* was performed using a PCR-based strategy based on the QuikChange® method (Stratagene) using the PHUSION polymerase (NEB). *S. cerevisiae* was transformed using Lithium-Acetate (Ito et al., 1983).

Molecular cloning
Plasmids (Table 1) were generated and modified using oligonucleotides listed in Table 3. In order to generate a minimal sense-and-response construct (${}^{ZIP\text{-}MBP}Mga2^{950\text{-}1062}$), the C-terminal region of *MGA2* containing juxtamembrane region (G950-S1113) and the predicted TMH was cloned into the pMAL-C2x-TEV expression vector via *Eco*RI/*Hind*III restriction sites. The resulting construct was truncated downstream of the TMH by introducing two consecutive stop codons after the residue at position 1062 by the PCR-based QuikChange® method. The *GCN4*-derived leucin zipper and a flexible linker $(GGGS)_2$ were introduced N-terminally to the MBP by restriction-based cloning using *Nde*I. Further mutagenesis of this construct and yeast expression vectors was performed either via the QuikChange® or the Q5 mutagenesis approach as indicated in Table 3.

Preparation of cell extracts and immunoblot analysis
Crude cell lysates were prepared as described previously [1] with minor modifications. Shortly, 15 OD600 equivalents of cells grown to the mid-exponential phase (OD600 ≈ 1.0) were harvested by centrifugation, washed with phosphate-buffered saline (PBS) supplemented with 10 mM NEM and snap-frozen. The cells were resuspended in 0.5 ml lysis buffer (PBS, 10 mM NEM, 5 mM EDTA, 10 µg/ml chymostatin, 10 µg/ml antipain, 10 µg/ml pepstatin) and lysed by bead-beating twice with 200 *µ*l zirconia beads (Roth) using a Scientific Industries SI™ Disruptor Genie™ Analog Cell Disruptor for 5 min each at 4 °C and 1 min pause on ice. For protein denaturation the extract was mixed at a ratio of 2:1 with 5x reducing sample buffer (8 M urea, 0.1 M Tris-HCl pH 6.8, 5 mM EDTA, 3.2% (w/v) SDS, 0.15% (w/v) bromphenol blue, 4% (v/v) glycerol, 4% (v/v) β-mercaptoethanol) and incubated at 60°C for 10 min.

Centrifugation (1 min, 16,000x g, room temperature) cleared protein samples were subjected to a discontinuous SDS-PAGE using 4-15% Mini-PROTEAN-TGX gels (BioRad). After semi-dry Western-Blotting onto nitrocellulose membranes, the target proteins were detected using specific antibodies. A list of antibodies, their used dilutions and source can be found in Supplementary Table 4.

Yeast growth assays / rescue of UFA auxotrophy

The UFA auxotroph Δ*SPT23*Δ*MGA2* strain was generated by Harald Hofbauer (Graz University) and cultivated in SCD-medium supplemented with 0.05% sodium linoleate. The cells were harvested by centrifugation, washed successively with 1% NP40-type tergitol (NP40S Sigma), then ddH_2O and then resuspended in SCD medium lacking any additives to an OD_{600} of 0.2. The cells were either cultivated at 30°C for 5-6 h to starve cells for UFAs prior to perform spotting tests or for 24 h to study the impact of mutations on the final cell density in liquid culture. For spotting tests, the UFA-starved cells were harvested and adjusted to an OD_{600} of 1. Serial 1:10 dilutions were prepared (10^0, 10^{-1},10^{-2}, 10^{-3}) and 5 μl of each dilution were spotted onto selective agar plates. The plates were incubated for 2-3 days at 30°C until sufficient cell growth became apparent.

The impact of linoleate on the final cell density in liquid medium was tested with UFA-depleted cultures that were adjusted to an OD_{600} of 0.05. 50 μl of these cultures were added to 180 μl SCD-Ura containing 1% NP40-type tergitol and varying concentrations of linoleic acid. The optical density of the cultures was determined using a microplate reader at 600 nm (OD_{600}) after 17 h of cultivation at 30°C.

Preparation of yeast cytosol

500 OD equivalents were harvested by centrifugation (5 min, 3000x g), washed with 30 ml ice cold PBS, then with 30 ml cold ubiquitylation buffer. The supernatant was decanted, and the cell pellet was resuspended in the residual liquid by vigorous vortexing. The resulting suspension was subjected dropwise into a tube with liquid nitrogen. The frozen beads of cells crushed with mortar and pistil (4 x 60 s and 1 x 90 s) and the resulting yeast powder was transferred into a cold 50 ml tube. The tube was immersed in water at room temperature and the thawing suspension was quickly adjusted to 1 mM DTT. Unbroken cells and debris were removed from the ice-cold suspension by centrifugation (10 min, 20,000x g, 4 °C). The supernatant of this step was centrifuged again (1 h, 100,000x g, 4 °C) to obtain the soluble, cytosolic fraction from the supernatant.

Expression, purification and labeling of ^{MBP}Mga2-fusions

Plasmids (Table 1) were used for the heterologous production of sensor construct in *E. coli*. The minimal sensor construct ($^{MBP}Mga2^{1032-1062}$) comprising the residues R1032-D1062 that include the TMH region of Mga2 was described previously [1]. The sense-and-response construct ($^{MBP}Mga2^{950-1062}$) was generated by cloning the coding regions of the JM and TMH region of Mga2 (residues 950-1062) into the pMal-C2x vector. The $^{ZIP-MBP}Mga2^{950-1062}$ construct was generated by fusing the leucine zipper sequence derived from the *GCN4* transcription factor (residues 249-281) in frame to MBP protein. The minimal sensor construct and the sense-and-response construct were overexpressed in the cytosol of *E. coli* BL21(DE3)pLysS and isolated essentially as described previously [1,2] with minor modifications. A 500 ml culture in LB_{rich} medium (LB medium supplemented with 2% glucose, 100 mg/ml ampicillin, 34 µg/ml

chloramphenicol) was inoculated 1:50 using an overnight culture and cultivated at 37°C until an OD_{600} of ~0.6 was reached. Then, protein production was induced by isopropyl-β-D-thiogalactopyranoside (IPTG) at a final concentration of 0.3 mM. After 3 h of cultivation at 37 °C the cells were harvested by centrifugation and washed with PBS. For isolation of the proteins, the cells were resuspended in 40 ml of lysis buffer (50 mM HEPES pH 7.0, 150 mM NaCl, 1 mM EDTA, 10 µg/ml chymostatin, 10 µg/ml antipain, 10 µg/ml pepstatin, 2 mM DTT, 5 U/ml Benzonase) per liter of culture and disrupted by sonification using a SONOPULS HD2070 ultrasonic homogenizer (Bandelin) (4x 30s, power 30%, pulse 0.7 sec/0.3 sec). The protein was solubilized by gentle agitation in the presence of 50 mM β-Octylglucoside (β-OG) for 20 min at 4 °C. Non-solubilized material was pelleted by centrifugation (30 min, 100,000 x g, 4° C) and the supernatant was applied to washed and equilibrated amylose beads (NEB) using 6 ml of slurry per liter of culture. After binding (20 min at 4 °C) to the amylose column and washing the column with 26 column volumes (CV) wash buffer (50 mM HEPES pH 7.0, 200 mM NaCl, 1 mM EDTA, 50 mM β-OG) the protein was either labeled or directly eluted. The labeling of the proteins at single cysteine residues with 1 mM MTS (methanethiosulfonate) (Enzo Life Sciences) or 1 mM ATTO488/ATTO590 dyes (ATTO TEC GmbH) was performed on the amylose column during an overnight incubation at 4 °C including gentle shaking. This step was skipped for the isolation of unlabeled proteins. The fusion protein was eluted with elution buffer (50 mM HEPES pH 7.0, 150 mM NaCl, 1 mM EDTA, 10 mM maltose, 50 mM β-OG). The sense-and-response construct ($^{ZIP\text{-}MBP}$Mga2$^{950\text{-}1062}$) was further purified by preparative SEC using a Superdex 200 10/300 increase column in SEC-buffer (50 mM HEPES pH 7.0, 150 mM NaCl, 1 mM EDTA, 50 mM β-OG). The purified proteins could be stored at -80°C for extended periods of time in storage buffer (40 mM HEPES pH 7.0, 120 mM NaCl, 0.8 mM EDTA, 40 mM β-OG, and 20% (v/v) glycerol).

The efficiency of spin-labeling was determined for each construct by double-integration of the EPR resonances and a comparison to the signal of a 100 µM MTS standard. The determined spin-label concentration was put into relation to the protein concentration determined by absorption spectroscopy at A_{280}. The labeling efficiency for W1042C^{MTS} was > 95%.

The efficiency of labeling with fluorescent dyes was determined by absorption spectroscopy using the following extinction factors: 9.58*10^4 l mol^{-1} cm^{-1} (unlabeled protein K983 or K969), 9*10^4 l mol^{-1} cm^{-1} (ATTO488), 1.2*10^5 l mol^{-1} cm^{-1} (ATTO 590) and the correction factors were 0.1 for ATTO488 and 0.44 for ATTO590 according to the manufacturer's specification. Maximal absorption intensities were determined at 505 nm (ATTO488) or 597 nm (ATTO590). The labeling efficiency was ~60% (K983$^{ATTO\ 488}$) and ~90% (K969$^{ATTO\ 590}$).

Liposome preparation

Liposomes of defined compositions were generated by mixing 1,2-dioleoyl-*sn*-glycero-3-phosphocholine (DOPC), 1-palmitoyl-2-oleoyl-*sn*-glycero-3-phosphocholine (POPC), 2-dipetroselenoyl-sn-glycero-3-phosphocholine (18:1 (Δ6-*cis*)PC), 2-dielaidoyl-*sn*-glycero-3-phosphocholine (*trans*DOPC) or 1-palmitoyl-2-oleoyl-*sn*-glycero-3-phosphoethanolamine (POPE) from 20 mg/ml stocks, dissolved in chloroform to obtain following molar compositions: 1) 100% DOPC; 2) 50% DOPC, 50% POPC; 3) 25% DOPC, 75% POPC; 4) 100% POPC; 5) 100% (18:1 (Δ6-*cis*))PC; 6) 100% *trans*DOPC; 7) 100% (16:1 (Δ9-*trans*))PC; 8) 50% DOPC, 30% POPC, 20% POPE; 9) 50% DOPC, 10% POPC, 40% POPE. After evaporation of the organic solvent using a constant stream of nitrogen, the lipid film was dried in a desiccator under vacuum (2 – 4 mbar) for at least 1 h at room temperature. For rehydration, the lipid film

was resuspended in reconstitution buffer (20 mM HEPES, pH 7.4, 150 mM NaCl, 5% (w/v) glycerol) to a final lipid concentration of 10 mM, incubated at 60 °C under rigorous shaking for 30 min at 1200 rpm, and incubated in a sonication in a water bath at 60°C for 30 min. The resulting multilamellar liposomes were used for reconstitution experiments.

Reconstitution of ^{MBP}Mga2-fusions in proteoliposomes

For reconstitution of the $^{ZIP\text{-}MBP}$Mga2$^{950\text{-}1062}$ constructs at a protein:lipid molar ratio of 1:5,000 - 1:15,000, 0.1 μmol lipid and 0.2 – 0.067 nmol protein were mixed in reconstitution buffer (20 mM HEPES (pH 7.4), 150 mM NaCl, and 5% (w/v) glycerol), adjusted to 37 mM β-OG in a total volume of 1 ml and incubated for 20 min at room temperature under gentle agitating. For detergent removal, 500 mg of Bio-Beads™ SM-2 Adsorbent Media (BioRad) were added and the resulting mixture was incubated and gently mixed for 120 min at room temperature. The suspension was then transferred to a fresh tube containing 100 mg Bio-Beads™ SM-2 Adsorbent Media and further incubated for 60 min. 0.8 ml of the proteoliposome containing suspension was mixed with 2.2 ml Harvesting buffer (20 mM HEPES, pH 7.4, 75 mM NaCl). Proteoliposomes were harvested by centrifugation (200,000x g, 4 °C, 18 h) and resuspended either in the respective assay buffer.

DPH anisotropy

Liposomes of different lipid compositions were generated by consecutive extrusions through 400 nm and 200 nm filters (21 strokes each) in a LiposoFast (Avestin) extruder. The concentration of lipids was then adjusted to 0.1 mM with PBS. Diphenylhexatriene (DPH) was added to a final concentration of 0.5 μM. The samples were incubated for 20 min in the dark at room temperature. Intensities for each polarized component (I_{hv}, I_{hh}, I_{vv}, I_{vh}) were recorded on a FluoroMax-4 spectrofluorometer using the following settings. The sample was excited at 360 nm and the emission was recorded at 430 nm with slit widths of 5 nm. A maximum of 10 measurments were performed with standard deviation cutoff of 5 %. The samples were equilibrated for 5 min for each temperature. To correct for scattered light, the intensities for each polarized component of the respective liposome sample before the addition of DPH were subtracted. The G factor and anisotropy (r) were calculated as follows $G = \frac{I_{hv}}{I_{hh}}$, $r = \frac{I_{vv} - G^{*} I_{vh}}{I_{vv} + 2^{*} G^{*} I_{vh}}$.

Thin layer chromatography

For lipid extraction, a 60 μl sample of $^{ZIP\text{-}MBP}$Mga2$^{950\text{-}1062}$-containing proteoliposomes reconstituted at a protein to lipid ratio of 1:8,000 were used. The sample was mixed with 1 ml of $CHCl_3$:MeOH (2:1) and an artificial upper phase of 200 μl (48:47:3) MeOH:H_2O:$CHCl_3$ and constantly agitated for 2 h at 4°C. After centrifugation (3,000 x g, 4°C, 5 min) the aqueous phase was discarded. The organic solvent evaporated from the remaining sample under a constant stream of N_2. Residual traces of the solvent were removed in 30 min using a desiccator and by applying vacuum. The extracted lipids were then taken up in 15 μl $CHCl_3$:MeOH (2:1). 1 μl of the extract was spotted onto an HPTLC Silica gel 60 (Merck KGaA) and separated using as a mobile phase (97.5:37.5:6) $CHCl_3$:MeOH:H_2O. As a reference, 1 μl of POPC, DOPC and POPE at a concentration of 20 mg/ml in $CHCl_3$ were spotted onto the plate. Additionally, a buffer control (50 mM Hepes pH 7.4, 150 mM NaCl, 5% w/v glycerol) was used. The silica plates were stained for 30 min using iodine.

Sucrose density gradient centrifugation

For validation of the reconstitution procedure, 200 µl of a proteoliposomal preparation were mixed with 400 µl 60% (w/v) sucrose solution in reconstitution buffer and overlaid with different layers of distinct densities. For protein-to-lipid molar ratios of 1:5,000, 1:8,000, and 1:15,000, the proteoliposome-containing layer was overlaid with each 2.5 ml of 20%, 10%, 5% and 0% (w/v) sucrose in reconstitution buffer. After centrifugation (100,000x g, 4°C, overnight) the gradient was fractionated from top to bottom in 13 fractions of 0.85 ml each. The distribution of the MBP-containing fusion proteins in the gradient was analyzed by SDS-PAGE and subsequent immunoblotting. The lipid content of the individual fractions was estimated by adjusting each fraction to 7 µM Hoechst 33342 and determination of the fluorescence intensity using a TECAN microplate reader (ex355 nm: em459, bandwidth 20 nm).

Recording and analysis of cwEPR spectra

cwEPR spectra were recorded and analyzed as previously described[1].

Isolation of ^{His}ubiquitin

^{8xHis}ubiquitin was overproduced in *E. coli* BL21(DE3)pLysS and purified using immobilized metal affinity chromatography (Ni^{2+}-NTA matrix). The plasmid encoding the human ubiquitin with an N-terminal 8xHis-tag was derived from a pETM-m60 plasmid and kindly provided by the Volker Dötsch lab. The production of ^{8xHis}ubiquitin was induced at an OD_{600} of ~0.6 at 37 °C using 0.3 mM IPTG. After induction, the cells were cultivated for additional 3 h at 30 °C prior to harvesting and washing of the cell pellet using PBS. For purification, the cells were resuspended in 20 ml lysis buffer (50 mM HEPES, pH 8.0, 250 mM NaCl, 20 mM imidazol, 10 µg/ml chymostatin, 10 µg/ml antipain, 10 µg/ml pepstatin) and disrupted by sonification (3x 30s, power 30%, pulse 0.7 s/ 0.3 s). Unbroken cells, debris, and cellular membranes were removed by centrifugation (1 h, 100,000x g, 4 °C). The cleared lysate was applied to 1 ml Ni^{2+}-NTA agarose matrix and incubated for 1 h at 4 °C while rotating to allow for protein binding. The mixture was then transferred into a gravity flow column and the flow-through was collected. The affinity matrix was washed with 30 CV of wash buffer (50 mM HEPES pH 8.0, 250 mM NaCl, 20 mM imidazole). ^{8xHis}ubiquitin was eluted with elution buffer (50 mM HEPES pH 8.0, 250 mM NaCl, 400 mM imidazole). The eluate was dialysed against 100-fold volume storage buffer (50 mM HEPES, pH 7.4, 150 mM NaCl) using a dialysis membrane with a molecular weight cutoff of 3.5 kDa (Spectra/Por). After 2 h the storage buffer was refreshed, and the sample was dialyzed overnight at 4°C. For long-term storage, the purified ^{8xHis}ubiquitin was adjusted to 1 mg/ml and 20% (w/v) glycerol in storage buffer.

Molecular dynamics simulations

We performed coarse-grained simulations in the MARTINI v2.2 force field [3,4]. We modelled TMHs containing the mutations W1042F, W1042Q, and W1042Y with the UCSF Chimera package[5] (Pettersen et al., 2004), and coarse grained in MARTINI[4]. For each mutation, we inserted two identical TMHs in a POPC lipid bilayer spanning the periodic simulation box in the xy-plane. We obtained simulation boxes containing two TMHs surrounded by approximately 560 lipids, 9000 water beads, and 150 mM sodium chloride. After energy minimization and equilibration, we ran 10 independent MD simulations for each system, totaling a simulated time of 1 ms for the W1042F and W1042Q systems each, and 0.93 ms for W1042Y one. All

simulations were performed in GROMACS 4.6.7, using a time step of 20 fs. A temperature of 303 K and a pressure of 1 atm were maintained with the velocity rescaling thermostat[6] and the semiisotropic Parrinello-Rahman barostat[7].

All-atom simulations of empty bilayers were performed with NAMD[8], using the CHARMM36 force field parameters for lipids[9,10]. Four bilayer systems were constructed and simulated as indicated in Supplementary Table 5. For the POPC trajectory, we used data from a previous simulation that contained 64 lipids per leaflet, 45 water molecules per lipid and no ions[11]. All bilayers were simulated at constant temperature of 30°C and constant pressure of 1 atm with the same simulation parameters as previously used[11].

Each trajectory was centered so that the geometric center of all terminal methyl carbons of the lipids was at (x,y,z)=(0,0,0). The last ~270 ns were used for analysis with frames being output every 20 ps. The average area per lipid (APL) was calculated by dividing the lateral area of the simulation box by the number of lipids in one leaflet. Thickness was calculated as the mean distance between the average z position of the phosphate atoms in each leaflet. The errors on both APL and thickness represent the standard deviation of the time series of the respective property.

Acyl chain order parameters were computed with an in-house tcl script. The order parameter S_{CD} at a carbon position is expressed as $S_{\mathrm{CD}} = \frac{1}{2}\langle 3\cos^2\theta - 1\rangle$ where $\langle\cdot\rangle$ denotes ensemble average and θ is the angle between a CH bond at that carbon and the bilayer normal (assumed to be the z dimension of the simulation box).

Local number density of lipid atoms was calculated with the volmap plugin in VMD[12]. A fixed region of the bilayer between -23 Å and 23 Å in each dimension was divided into grid points spaced 1×1×1 Å apart. Atoms were represented as normalized gaussians with standard deviation equal to the atom radius. This representation was used to calculate their atomic number density at each grid point. The atomic radii used in the calculation were the default atomic radii in VMD, which mimic the atomic radii in the CHARMM36 force field parameters: carbon 1.5 Å, hydrogen 1.0 Å, nitrogen 1.4 Å, oxygen 1.3 Å. The densities calculated at the grid points were subsequently analyzed with MATLAB to produce the density profiles as a function of z and the heatmap representations in which the data was collapsed on the xz plane by taking the average across all corresponding y values.

Lateral pressure profiles were calculated from the last 230-250 ns of the trajectories with NAMD as previously explained[11]. Each profile was symmetrized by means of averaging the pressure profiles of the two leaflets (i.e. above and below z=0) and smoothed with a 7-point moving average window in MATLAB. Bilayer snapshots of the all-atom bilayers were rendered with VMD.

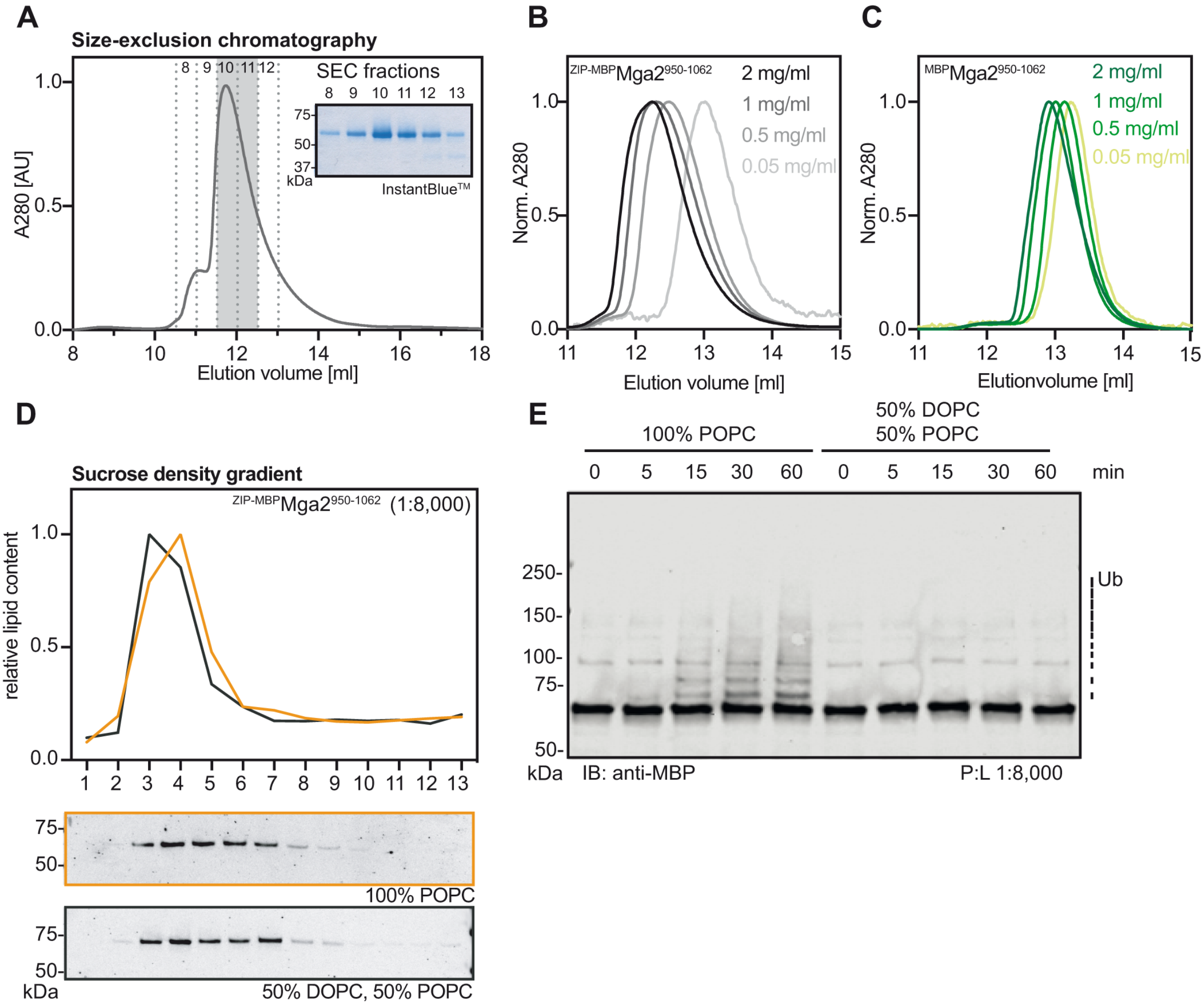

Supplementary Figure 1. Isolation and functional reconstitution of sense-and-response construct.

(**A**) Purification of the zipped sense-and-response construct ($^{ZIP\text{-}MBP}Mga2^{950\text{-}1062}$) by SEC. The eluate of the affinity purification (Figure 2B) was concentrated ~10fold and loaded onto a Superdex 200 10/300 Increase column (void volume 8.8 ml) using a 500 μl loop. Fractions of 0.5 ml were collected, mixed with non-reducing membrane sample buffer and subjected to SDS-PAGE followed by InstantBlue™ staining. Fraction 10 and 11 were pooled and further used. (**B**) SEC of the purified $^{ZIP\text{-}MBP}Mga2^{950\text{-}1062}$ protein in the detergent-containing SEC-buffer. The protein concentration was adjusted to the indicated concentrations, and 100 μl of each of these samples were subjected to SEC using a Superdex 200 10/300 Increase column. (**C**) SEC of the purified non-zipped $^{MBP}Mga2^{950\text{-}1062}$ protein in SEC-buffer. The protein concentration was adjusted to the indicated concentrations, and 100 μl of each of these samples were loaded onto a Superdex 200 10/300 Increase column. (**D**) Sucrose-density gradients centrifugation for proteoliposomes containing $^{ZIP\text{-}MBP}Mga2^{950\text{-}1062}$ at a molar protein:lipid ratio of 1:8,000. The proteoliposome sample was adjusted to 40% w/v sucrose and overlaid with sucrose cushions of different concentrations (20%, 10%, 5%, 0% w/v). After ultracentrifugation, 13 fractions were collected from top to bottom. The relative content of lipids in the individual fractions was determined by Hoechst 33342 fluorescent staining. The amount of $^{MBP}Mga2$-TMH in the fractions was monitored by immunoblotting using anti-MBP antibodies. (**E**) *In vitro* ubiquitylation reactions were performed with the WT $^{ZIP\text{-}MBP}Mga2^{950\text{-}1062}$ sense-and-

response construct reconstituted in the indicated lipid environments at a protein:lipid ratio of 1:8,000. After indicated times, the reactions were stopped and subjected to SDS-PAGE. For analysis, an immunoblot using anti-MBP antibodies was performed. Source data are provided as a Source Data file.

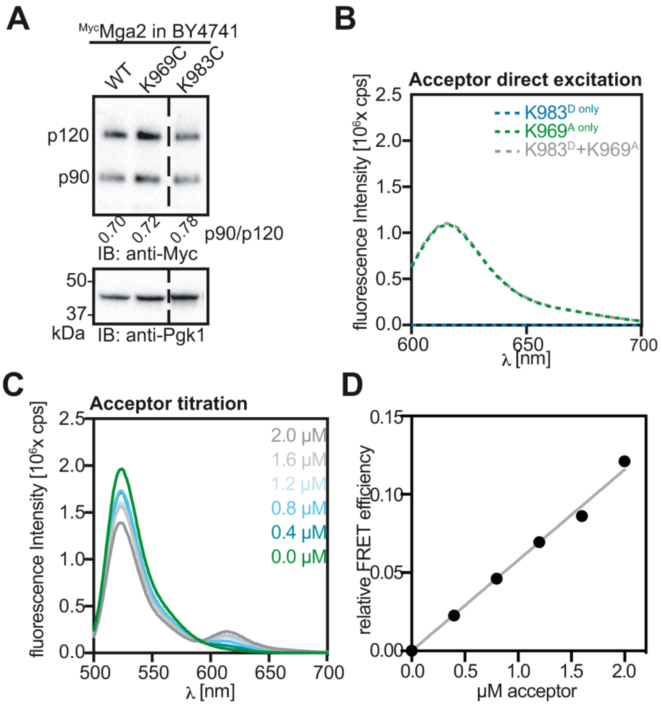

Supplementary Figure 2: Establishing a FRET reporter based on sense-and-response construct.

(**A**) Immunoblot analysis of indicated ^{Myc}Mga2 variants produced at near-endogenous levels in the BY4741 wild type background. Cells were cultivated in YPD to the mid-logarithmic growth phase. Crude cell lysates were subjected to SDS-PAGE and analyzed by immunoblotting using anti-Myc antibodies. The Mga2 p90:p120 ratios were determined by densiometric quantification. An anti-Pgk1 immunoblot served as loading control. (**B**) Fluorescence emission spectra for the samples in shown in Figure 3B upon direct acceptor excitation at 590 nm). (**C**) 2 μM donor was titrated with the indicated acceptor concentrations and fluorescence emission spectra were measured upon donor excitation. The overall protein concentrations were maintained by the use of unlabeled $^{ZIP\text{-}MBP}$Mga2$^{950\text{-}1062}$. (**D**) Relative FRET efficiencies were determined from the donor/acceptor intensity ratios in (C). Data were fitted via linear regression. Source data are provided as a Source Data file.

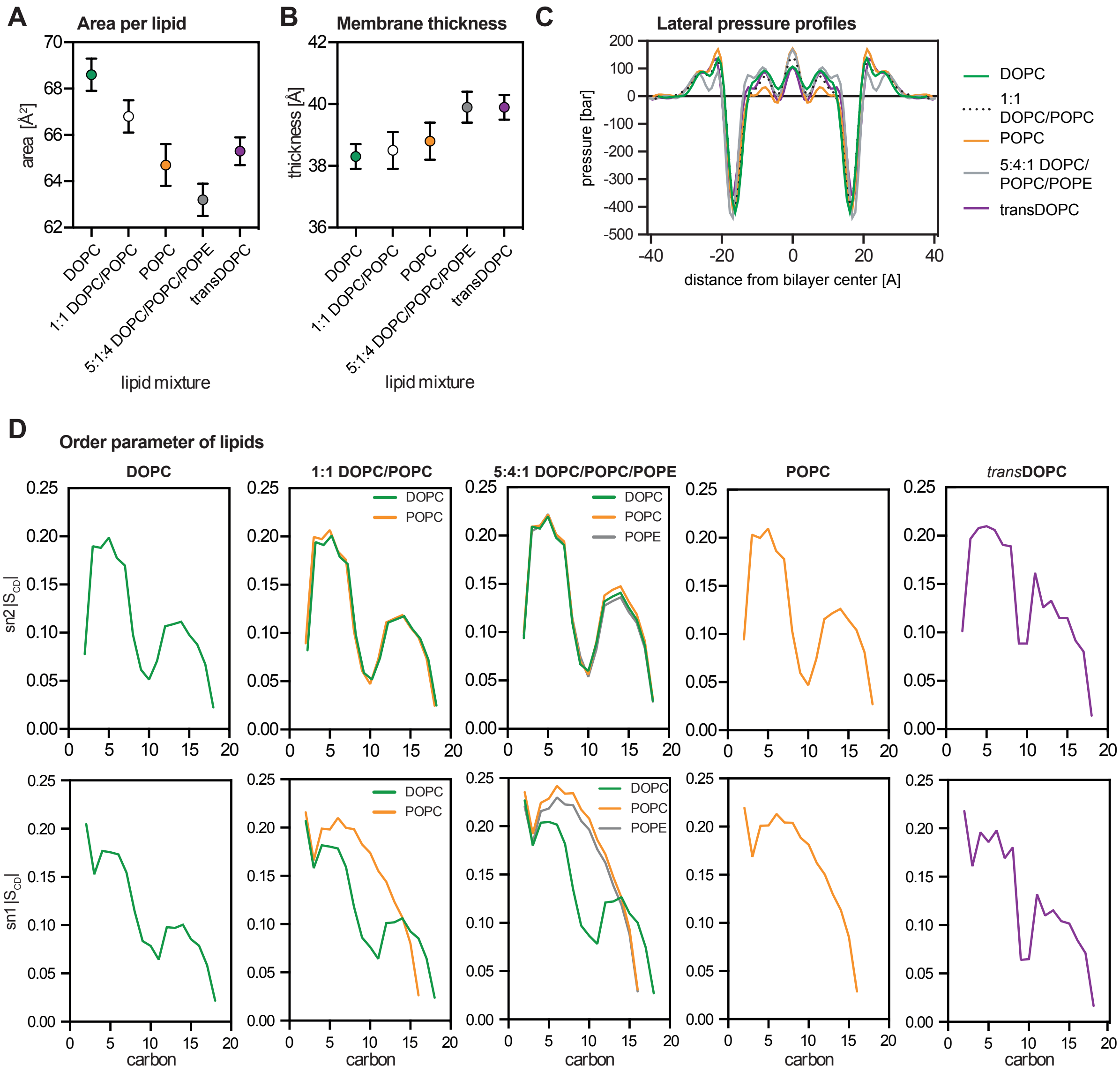

Supplementary Figure 3: Biophysical properties of lipid bilayers determined from all-atom simulations. (A) Plotted is the average area per lipid and (**B**) the phosphate-to-phosphate thickness in different bilayer systems. Acyl chain saturation and the presence of PE lipids increase lipid packing (decrease area per lipid) and have a more modest effect on bilayer thickness. **(C)** Lateral pressure distribution in the bilayers as a function of distance from the bilayer center. Acyl chain saturation increases the pressure at the bilayer midplane while in the region of the sensory W1042 residue of Mga2 the pressure is similar in all bilayers except for POPC where it is lower. **(D)** Acyl chain order parameter of each lipid type in the different bilayers. Minimal changes are observed in the oleoyl *sn*-2 chain of the lipids while more pronounced differences consistent with the changes in lipid packing (A) can be seen in the order parameter of the lipids' *sn*-1 chain. Source data are provided as a Source Data file.

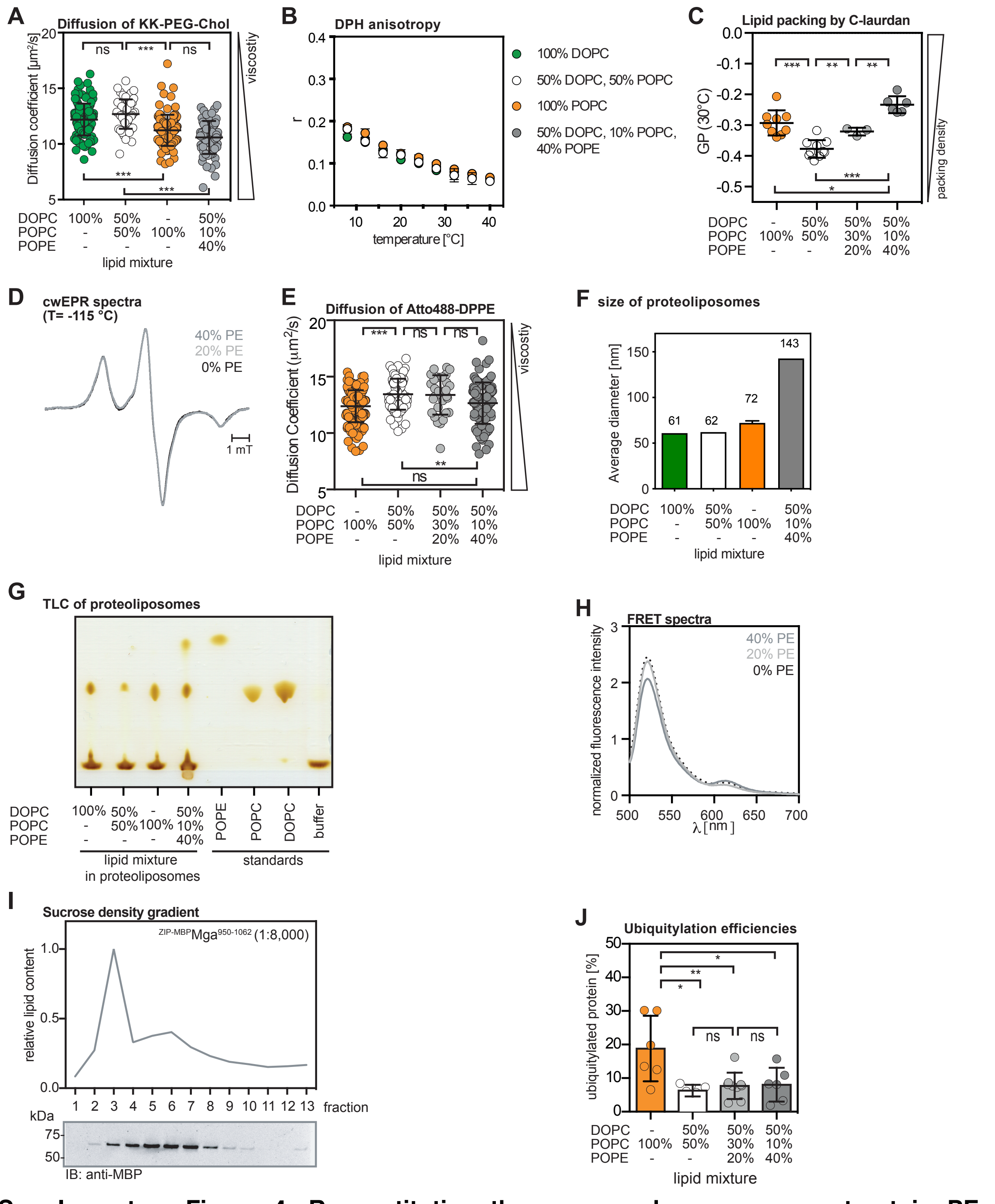

Supplementary Figure 4: Reconstituting the sense-and-response construct in PE-containing liposomes.

(**A**) Diffusion coefficients of Star Red-PEG Cholesterol in giant unilaminar vesicles of the indicated lipids were determined by confocal point-FCS. Plotted is the mean ± SD (n_{DOPC}=127, $n_{(1:1)\ DOPC:POPC}$=53, n_{POPC}=110, $n_{40\%\ POPE}$=66). A Kolmogorov-Smirnov test was performed to test for statistical significance (***p<0.001). (**B**) The anisotropy of DPH was determined at different temperatures and in liposomes with the indicated compositions. The data are plotted as the mean ± SD of three independent experiments. (**C**) The lipid packing in liposomes composed of DOPC:POPC:POPE at a ratio of 5:3:2 was determined via C-Laurdan spectroscopy at 30°C. The GP values shown for POPC, POPC:DOPC at a ratio of 1:1, and

DOPC:POPC:POPE at a ratio of 5:1:4 are identical to the data in Figure 4B. The data are plotted as mean ± SD (n_{POPC}=9; $n_{(1:1)DOPC:POPC}$=10; $n_{20\%PE}$=3; $n_{40\%PE}$=6). An unpaired two-tailed, students t-test was performed to test for statistical significance (*p<0.05, **p<0.01, ***p<0.001). (**D**) Intensity normalized cwEPR spectra recorded at -115°C for a fusion protein composed of MBP and the TMH of Mga2 (^{MBP}Mga2$^{1032\text{-}1062}$) labeled at position W1042C was reconstituted at a molar protein:lipid of 1:500 in liposomes composed of the indicated lipid mixtures. (**E**) Diffusion coefficients of the fluorescent lipid analogue Atto488-DPPE in giant unilaminar vesicles containing DOPC:POPC:POPE at a molar ratio of 5:3:2 were determined by confocal point FCS. The plotted diffusion coefficients of Atto488-DPPE in liposomes composed of either POPC, POPC:DOPC at molar ratio of 1:1, or DOPC:POPC:POPE at a molar ratio of 5:4:1 are the same as in Figure 4B. The data are represented as mean ± SD (n_{DOPC}=172; $n_{(1:1)DOPC:POPC}$=81; n_{POPC}=153; $n_{20\%PE}$= 30 $n_{40\%PE}$=100). A Kolmogorov-Smirnov test was performed to test for statistical significance (***p<0.001). (**F**) The average diameter of the proteoliposomes containing the FRET reporter and with the indicated lipid composition was determined by dynamic light scattering (Malvern Zetasizer Nano S90) and plotted. The experiments were performed only once with proteoliposomes from a reconstitution generating membrane environments with DOPC, POPC:DOPC at a molar ratio of 1:1, and DOPC:POPC:POPE at a molar ratio of 5:4:1. They were performed for two independent reconstitutions generating a POPC membrane environment. (**G**) Thin layer chromatography (TLC) of $^{ZIP\text{-}MBP}$Mga2$^{950\text{-}1062}$-containing proteoliposomes with the indicated lipid composition. Lipids were extracted from the proteoliposomes, spotted onto a HPTLC Silica gel 60 plate, and separated using 97.5:37.5:6 $CHCl_3$:MeOH:H_2O as a mobile phase prior to iodine staining. As controls served 1 μl of the indicated lipid stocks at a concenration of 20 mg/ml in $CHCl_3$ or reconstitution buffer (50 mM Hepes pH 7.4, 150 mM NaCl, 5% w/v glycerol). (**H**) Fluorescence emission spectra of the (K983^{D}+K969^{A}) FRET pair reconstituted in liposomes composed of the indicated lipid mixtures were recorded (ex: 488 nm, em: 500-700 nm), normalized to the maximal acceptor emission after direct acceptor excitation (ex: 590 nm), and plotted. The emission spectra were normalized to acceptor emission after direct acceptor excitation. (**I**) Sucrose-density gradient centrifugation for proteoliposomes containing $^{ZIP\text{-}MBP}$Mga2$^{950\text{-}1062}$ at a molar protein:lipid ratio of 1:8,000 in a lipid mixture of 50 mol% DOPC, 10 mol% POPC and 40 mol% POPE. Samples were adjusted to 40% sucrose and overlaid with decreasing concentrations of sucrose-solution (20%, 10%, 5%, 0%). After ultracentrifugation 13 fractions were recovered from top to bottom. The relative content of lipids in the individual fractions was determined by Hoechst 33342 fluorescent staining. The amount of ^{MBP}Mga2-TMH in the fractions was monitored by immunoblotting using anti-MBP antibodies. (**J**) *In vitro* ubiquitylation of the zipped sense-and-response construct ($^{ZIP\text{-}MBP}$Mga2$^{950\text{-}1062}$) reconstituted in liposomes composed of 50% DOPC, 30% POPC, 20% POPE at a molar protein-to-lipid ratio of 1:8,000 were performed as described in the Supplementary Materials and analyzed by immunoblotting using anti-MBP antibodies. The signal intensities of ubiquitylated species were quantified using Image Studio Lite (LI-COR). Plotted is the mean ± SD ($n_{(POPC)}$=5; $n_{(50:50)}$= 5; $n_{(+20PE)}$= 7; $n_{(+40PE)}$= 5). Unpaired, two-tailed t-test were performed to test for statistical significance (*p<0.05, **p<0.01). Source data are provided as a Source Data file.

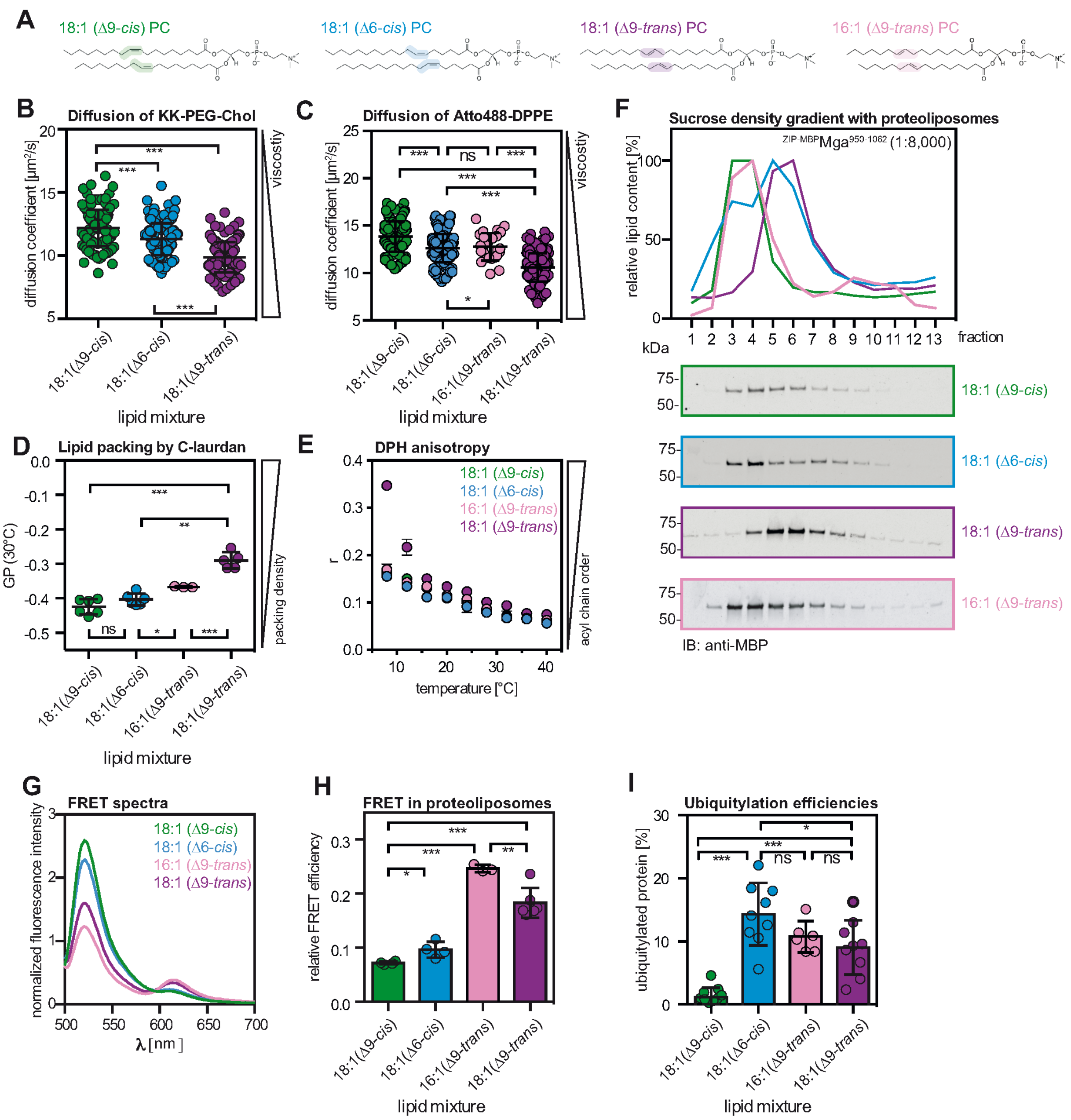

Supplementary Figure 5: Reconstituting the sense-and-response construct in liposomes with different PC-species.

(**A**) Chemical structure of the four relevant PC lipids with distinct double bonds isomers and positions. All lipids contain a PC head group, two acyl chains of 18 or 16 carbons with one double bond. They differ in the position (Δ9 or Δ6) and the orientation of the double bond (*cis* or *trans*). The color code is maintained in (B-I). (Structures adapted from avantilipids.com) (**B**) Diffusion coefficients of Star Red-PEG Cholesterol in giant unilaminar vesicles of the indicated lipids were determined by confocal point FCS. Plotted is the mean ± SD ($n_{18:1(\Delta 9\text{-cis})}$= 127, $n_{18:1(\Delta 6\text{-cis})}$= 132, $n_{18:1(\Delta 9\text{-trans})}$= 132). A Kolmogorov-Smirnov test was performed to test for statistical significance (***p<0.001). (**C**) Diffusion coefficient of the fluorescent lipid analogue Atto488-DPPE in giant unilaminar vesicles composed of 16:1(Δ9-*trans*)PC was determined by confocal point FCS. The diffusion coefficients of Atto488-DPPE in DOPC (18:1(Δ9-*cis*), in PC lipids with two 18:1(Δ6-*cis*) acyl chains, and PE lipid with two 18:1(Δ9-trans) acyl chains are identical to the ones shown in Figure 5A. The data are plotted as mean ± SD ($n_{(18:1(\Delta 9\text{-}cis))}$=172; $n_{(18:1(\Delta 6\text{-}cis))}$=162; $n_{(16:1(\Delta 9\text{-}trans))}$=25; $n_{(18:1(\Delta 9\text{-}trans))}$=163). A Kolmogorov-Smirnov test was performed

to test for statistical significance (*p<0.05, ***p<0.001). (**D**) The lipid packing in liposomes composed of 16:1(Δ9-*trans*)PC was determined via C-Laurdan spectroscopy at 30°C. GP values of C-Laurdan in DOPC (18:1(Δ9-*cis*)), in PC lipids with two 18:1(Δ6-*cis*) acyl chains, and in PC lipids with two 18:1(Δ9-*trans*) are identical to the ones shown in Figure 5B. The data are plotted as mean ± SD ($n_{(18:1(\Delta 9\text{-}cis))}$= 6, $n_{(18:1(\Delta 6\text{-}cis))=}$ 6; $n_{(16:1(\Delta 9\text{-}trans))}$= 3, $n_{(18:1(\Delta 9\text{-}trans))}$= 5). An unpaired two-tailed, students t-test was performed to test for statistical significance (**p<0.01, ***p<0.001). (**E**) Anisotropy of DPH was determined at different temperatures and in liposomes with the indicated lipid compositions. The data are plotted as the mean ± SD of three independent experiments. (**F**) Sucrose-density gradient centrifugation of proteoliposomes of the indicated lipid composition containing $^{ZIP\text{-}MBP}$Mga2$^{950\text{-}1062}$ reconstituted at a molar protein:lipid ratio of 1:8,000. The proteoliposome samples were adjusted to 40% (w/v) sucrose and overlaid with four containing different concentrations of sucrose (20% (w/v), 10% (w/v), 5% (w/v), 0% (w/v)). After centrifugation (100,000x g, 4°C, overnight) the gradient was fractionated from top to bottom. The distribution of lipids and proteins in the gradient was determined as described in the Supplementary Materials. (**G**) The fluorescence emission spectrum of the (K983^{D}+K969^{A}) FRET pair reconstituted in liposomes composed of 16:1(Δ9-*trans*)PC lipids was recorded (ex: 488 nm, em: 500-700 nm) and plotted after normalization to the maximal emission upon direct excitation of the acceptor (ex: 590 nm). The data for DOPC (18:1(Δ9-*cis*)), PC lipids with either two 18:1(Δ6-*cis*) or two 18:1(Δ9-*trans*) acyl chains are identical to the data shown in Figure 5E. (**H**) The relative FRET efficiencies were calculated from fluorescence spectra as in (G). The data are plotted as the mean ± SD ($n_{(18:1(\Delta 9\text{-}cis))}$=4; $n_{(18:1(\Delta 6\text{-}cis))}$= 4; $n_{(16:1(\Delta 9\text{-}trans))}$= 3; $n_{(18:1(\Delta 9\text{-}trans))}$= 6). A two-tailed, unpaired t-test was performed to test for statistical significance (*p<0.05; **p<0.005). The data for DOPC (18:1(Δ9-*cis*)), 18:1(Δ6-*cis*)PC and 18:1(Δ9-*trans*)PC are identical to the one in Figure 5F. (**I**) *In vitro* ubiquitylation of the zipped sense-and-response construct ($^{ZIP\text{-}MBP}$Mga2$^{950\text{-}1062}$) reconstituted in liposomes composed of 16:1(Δ9-*trans*)PC at a molar protein-to-lipid ratio of 1:8,000 were performed as described in the Supplementary Materials. After the reaction was stopped, the samples were subjected to SDS-PAGE and analyzed by immunoblotting using anti-MBP antibodies. The signal intensities of ubiquitylated species were quantified using Image Studio Lite (LI-COR). Plotted is the mean ± SD ($n_{(18:1(\Delta 9\text{-}cis))}$=20; $n_{(18:1(\Delta 6\text{-}cis))}$= 9; $n_{(16:1(\Delta 9\text{-}trans))}$= 6; $n_{(18:1(\Delta 9\text{-}trans))}$= 9). The data for DOPC (18:1(Δ9-*cis*)PC), 18:1(Δ6-*cis*)PC and 18:1(Δ9-*trans*)PC are identical with the data in Figure 5H. Source data are provided as a Source Data file.

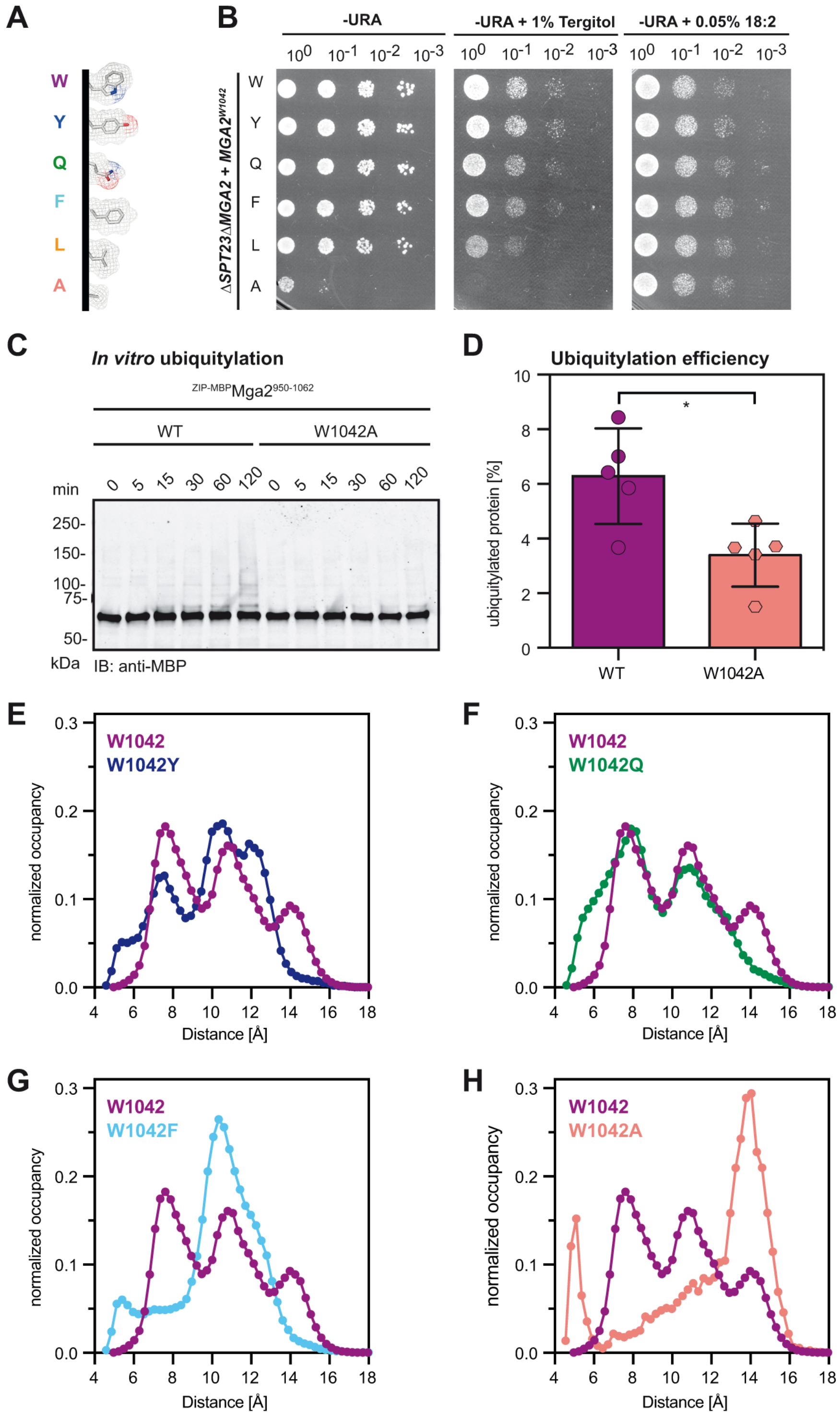

Supplementary Figure 6: Mutagenesis of sensory residue W1042 and phenotypic characterization.

(**A**) Representations of the amino acids (and substitutions) at position of the sensory W1042 in the TMH of Mga2. The side-chain structures were modeled using PyMOL and are shown as sticks with electron meshes. (**B**) Spotting test for rescue of UFA auxotrophy. The indicated *MGA2* variants were expressed from their endogenous promoters on *CEN*-based plasmids in the Δ*SPT23*Δ*MGA2* strain background. Cultures were cultivated in the absence of exogenously provided UFAs for 5 h and then spotted in a ten-fold dilution series on SCD-URA

plates with the indicated additives. The resulting colonies were documented after 2 days of cultivation at 30 °C. (**C**) *In vitro* ubiquitylation of the zipped sense-and-response construct $^{ZIP\text{-}MBP}$Mga2$^{950\text{-}1062}$ wild type (WT) and a W1042A variant reconstituted at a protein:lipid molar ratio of 1:15,000 in POPC. After the reaction was stopped, ubiquitylated species were detected by SDS-PAGE and subsequent immunoblotting using anti-MBP antibodies. (**D**) Densiometric quantification of the *in vitro* ubiquitylation assays as in (C). The fraction of ubiquitylated protein was determined for the indicated time points and for the wildtype (WT) and W1042A variant of the sense-and-response construct. Plotted is the mean ± SD (n=5). The statistical significance was tested by a two-tailed, unpaired t-test (*$p<0.05$). (**E-H**) Distribution of distances between the backbone beads of residue 1042 in the TMH dimer of wildtype Mga2 (W1042) and the indicated variants, calculated from coarse-grained MD simulations performed with a POPC bilayer using the MARTINI v2.2 force field[3,4]. Data for the wildtype Mga2 and the W1042A mutation are re-plotted from a previous study with permission from Elsevier[1]. The population of different conformational states is strongly affected by the side chain of the amino acid at the position of the sensory W1042. Notably, mutations with the most prominent impact of the structural dynamics (W1042F and W1042A) have also have the strongest impact on the processing efficiency of Mga2 as experimentally shown in Figure 6D. Source data are provided as a Source Data file.

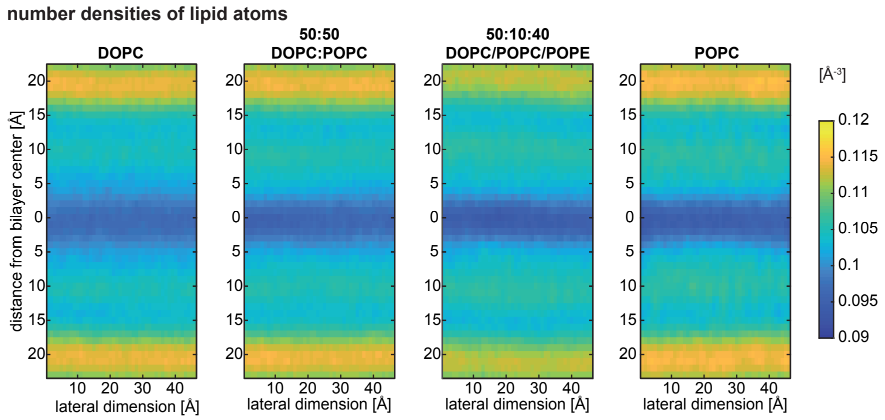

Supplementary Figure 7: The local number density of lipid atoms in different bilayer systems.

All-atom MD simulations were performed for the indicated bilayer systems. The number density of lipid atoms in cubic boxes with a side of length 1 Å was calculated and plotted as a heatmap. The x-axis shows the mean of the local densities in the x and y planes at the respective z position. Highest local number densities are indicated in yellow and observed in the region of the lipid headgroups. Lowest densities are indicated in dark blue and observed in the center of the lipid bilayer. Source data are provided as a Source Data file.

Supplementary Table 1. Plasmids used in this study.

Plasmid	Description	Source
in vivo		
pRS316	Empty vector (CEN6-ARS4, URA3, AMP)	EUROSCARF
pRE262	pRS316-3xMyc-*MGA2* WT	This study and [1]
pRE266	pRS316-3xMyc-*MGA2* W1042A	This study and [1]
pRE305	pRS316-3xMyc-*MGA2* W1042L	This study and [1]
pRE333	pRS316-3xMyc-*MGA2* W1042Y	This study
pRE334	pRS316-3xMyc-*MGA2* W1042F	This study
pRE335	pRS316-3xMyc-*MGA2* W1042Q	This study
pRE683	pRS316-3xMyc-*MGA2* K969C	This study
pRE684	pRS316-3xMyc-*MGA2* K983C	This study

Plasmid	Description	Source
in vitro		
pRE345	pMAL-C2x-MBP-*MGA2*-TMH W1042C	This study and [1]
pRE496	pETM-m60-8xHis-hUb WT	Provided by V. Dötsch
pRE714	pMAL-C2x-MBP-*MGA2*-JM-TMH WT	This study
pRE759	pMAL-C2x-ZIP-MBP-*MGA2*-JM-TMH WT	This study
pRE766	pMAL-C2x-ZIP-MBP-*MGA2*-JM-TMH ΔLPKY	This study
pRE767	pMAL-C2x-ZIP-MBP-*MGA2*-JM-TMH W1042A	This study
pRE771	pMAL-C2x-ZIP-MBP-*MGA2*-JM-TMH K980R, K983R, K985R	This study
pRE848	pMAL-C2x-ZIP-MBP-*MGA2*-JM-TMH K983C	This study
pRE849	pMAL-C2x-ZIP-MBP-*MGA2*-JM-TMH K969C	This study

Supplementary Table 2. Strains used in this study.

Strain Number	Description	Genotype	Source	Plasmid
ECRE01	E. *coli* DH5 alpha	F^- φ80*lac*ZΔ M15 Δ(*lac*ZYA-*arg*F) U169 *rec*A *end*A1 *hsd*R17(rK$^-$, mK$^+$) *pho*A *sup*E44 λ^- *thi*-1 *gyr*A96 *rel*A1	ThermoFisher EC0112	N/A
ECRE02	*E. coli BL21 Star™ (DE3)pLysS*	F-*omp*T hsdSB (rB$^-$, mB$^-$) *galdcmrne*131 (DE3) pLysS (Cam^R)	ThermoFisher C602003	pLysS
YRE001	BY4741	MATa; his3Δ1; leu2Δ0; met15Δ0; ura3Δ0	EUROSCARF[13] (Y00000)	N/A
YRE009	*ΔUBX2*	MATa; his3Δ1; leu2Δ0; met15Δ0; ura3Δ0; ubx2Δ::kanMX4	EUROSCARF[14] (Y00560)	N/A
YRE067	BY4741 3xMyc-*MGA2* WT	MATa; his3Δ1; leu2Δ0; met15Δ0; ura3Δ0	This study and [1]	pRE262
YRE068	BY4741 3xMyc-*MGA2* W1042A	MATa; his3Δ1; leu2Δ0; met15Δ0; ura3Δ0	This study and [1]	pRE266
YRE071	*ΔUBX2* 3xMyc-*MGA2* WT	MATa; his3Δ1; leu2Δ0; met15Δ0; ura3Δ0; ubx2Δ::kanMX4	This study and [1]	pRE262
YRE199	BY4741 3xMyc-*MGA2* W1042L	MATa; his3Δ1; leu2Δ0; met15Δ0; ura3Δ0	This study and [1]	pRE305
YRE216	BY4741 3xMyc-*MGA2* W1042Y	MATa; his3Δ1; leu2Δ0; met15Δ0; ura3Δ0	This study	pRE333
YRE217	BY4741 3xMyc-*MGA2* W1042F	MATa; his3Δ1; leu2Δ0; met15Δ0; ura3Δ0	This study	pRE334
YRE228	*ΔSPT23*, *ΔMGA2*	MATα; his3Δ1; leu2Δ0; lys2Δ0; ura3Δ0; spt23Δ::kanMX4; mga2Δ:natMX	This study Provided by H. Hofbauer	
YRE295	*ΔSPT23*, *ΔMGA2* 3xMyc-*MGA2* WT	MATα; his3Δ1; leu2Δ0; lys2Δ0; ura3Δ0; spt23Δ::kanMX4; mga2Δ:natMX	This study	pRE262
YRE296	*ΔSPT23*, *ΔMGA2* 3xMyc-*MGA2* W1042A	MATα; his3Δ1; leu2Δ0; lys2Δ0; ura3Δ0; spt23Δ::kanMX4; mga2Δ:natMX	This study	pRE266
YRE297	*ΔSPT23*, *ΔMGA2* 3xMyc-*MGA2* W1042L	MATα; his3Δ1; leu2Δ0; lys2Δ0; ura3Δ0; spt23Δ::kanMX4; mga2Δ:natMX	This study	pRE305
YRE404	BY4741 3xMyc-*MGA2* W1042Q	MATa; his3Δ1; leu2Δ0; met15Δ0; ura3Δ0	This study	pRE335
YRE415	BY4741 empty vector pRS316	MATa; his3Δ1; leu2Δ0; met15Δ0; ura3Δ0	This study	pRS316
YRE572	*ΔSPT23*, *ΔMGA2* 3xMyc-*MGA2* W1042Q	MATα; his3Δ1; leu2Δ0; lys2Δ0; ura3Δ0; spt23Δ::kanMX4; mga2Δ:natMX	This study	pRE335
YRE573	*ΔSPT23*, *ΔMGA2* 3xMyc-*MGA2* W1042F	MATα; his3Δ1; leu2Δ0; lys2Δ0; ura3Δ0; spt23Δ::kanMX4; mga2Δ:natMX	This study	pRE334
YRE574	*ΔSPT23*, *ΔMGA2* 3xMyc-*MGA2* W1042Y	MATα; his3Δ1; leu2Δ0; lys2Δ0; ura3Δ0; spt23Δ::kanMX4; mga2Δ:natMX	This study	pRE333
YRE578	*ΔSPT23*, *ΔMGA2* empty vector pRS316	MATα; his3Δ1; leu2Δ0; lys2Δ0; ura3Δ0; spt23Δ::kanMX4; mga2Δ:natMX	This study	pRS316

Supplementary Table 3. Oligonucleotides used for molecular cloning.

Name	Sequence (5'→ 3')	Description
SB1	GGGAATTCGGTAGTACATCTCTCTGGAATAGAGTTTTAC	GG-*Eco*RI-*MGA2* (bp 2974–2996) forward (f)
SB2	CCCAAGCTTCTAACTGACAATTAAATCGTTCAACATTC	CCC-*Hind*III-*MGA2* (bp 3437–3465) reverse (r)
RE337	GATAAAATGTTAATATTTTTCTTGATACCCTTAACACTACTAC	*MGA2* W1042L (f); QuikChange
RE338	GTAGTAGTGTTAAGGGTATCAAGAAAAATATTAACATTTTATC	*MGA2* W1042L (r); QuikChange
RE410	CCATCACGGTTCTGGTCAGATTTTCGTGAAAACCC	8xHis-*UB* tagging; Q5 (f)
RE411	TGATGGTGATGGTGATGCATGGTATATCTCCTTCTTAAAG	8xHis-*UB* tagging; Q5 (r)
RE925	GCAACATATGAGAACCACCACCGCGTTCGCCAACTAATTTCT	*Nde*I-SGGG-*GCN4* (ZIP) (r)
RE926	AGCGTCCGAGCATCATATGATGAGAATGAAACAACTTGAAGACAA	13 bp-*Nde*I-*GCN4* (ZIP) (f)
RE930	ATCGGAATTCGGTGGCGGTTCTGG	ATCG-*Eco*RI-$(GGGS)_2$-*MGA2* (bp3217-3233) (f)
RE931	ACGCAAGCTTTTAATCTTGGTTGCCAAATTTGTAC	ACGC-*Hind*III-*MGA2*-TMH (bp3311-3333) (r)
RE934	GCGGTGGTGGTTCTGGTGGAGGTTCTAAAATCGAAGAAGG	IntGGGS ZIP_MBP; QuikChange (f)
RE935	CCTTCTTCGATTTTAGAACCTCCACCAGAACCACCACCGC	IntGGGS ZIP_MBP; QuikChange (r)
RE252	CCGAAATGATAAAATGTTAATATTTTTCGCTATACCCTTAACACTACTACTTTTGACATGG	*MGA2* W1042A; QuikChange (r)
RE253	CCATGTCAAAAGTAGTAGTGTTAAGGGTATAGCGAAAAATATTAACATTTTATCATTTCGG	*MGA2* W1042A; QuikChange (f)
RE260	GAGGATCTGTTCCCGTTGTCTTGGGGTCGTGATGATCGTTTGCGTACCACAAATCAAGACAGTATTGTGGAGCAG	*MGA2* K980R,K983R, K985R; QuikChange (f)
RE261	CTGCTCCACAATACTGTCTTGATTTGTGGTACGCAAACGATCATCACGACCCCAAGACAACGGGAACAGATCCTC	*MGA2* K980R, K983R, K985R; QuikChange (r)
RE471	ACAAAACTTCCGAAATGATAAAATGTTAATATTTTTCTATATACCCTTAACACTACTACTTTTGAC	*MGA2* W1042Y; QuikChange (f)
RE472	GTCAAAAGTAGTAGTGTTAAGGGTATATAGAAAAATATTAACATTTTATCATTTCGGAAGTTTTGT	*MGA2* W1042Y; QuikChange (r)
RE473	AAAACTTCCGAAATGATAAAATGTTAATATTTTTCTTCATACCCTTAACACTACTACTTTTGA	*MGA2* W1042F; QuikChange (f)
RE474	TCAAAAGTAGTAGTGTTAAGGGTATGAAGAAAAATATTAACATTTTATCATTTCGGAAGTTTT	*MGA2* W1042F; QuikChange (r)
RE541	AATATTTTTCCAAATACCCTTAACACTACTACTTTTG	*MGA2* W1042Q; Q5 (f)
RE542	AACATTTTATCATTTCGGAAGTTTTG	*MGA2* W1042Q; Q5 (r)
RE603	GGCAACCAAGATTGATAAATCAATCATATAAGCG	*MGA2* S1063*, S1064*; QuikChange (f)
RE604	CGCTTATATGATTGATTTATCAATCTTGGTTGCC	*MGA2* S1063*, S1064*; QuikChange (r)
RE764	CGAAATGATAAAATGTTAATATTTTTCGCTATACCCTTAACACTACTACTTTTGAC	*MGA2* W1042A; QuikChange (f)
RE765	GTCAAAAGTAGTAGTGTTAAGGGTATAGCGAAAAATATTAACATTTTATCATTTCG	*MGA2* W1042A; QuikChange (r)
RE902	GTCATCATTAATTCGATGTAAAAC	ΔLPKY ^{MBP}Mga2-LPKY-TMH; Q5 (f)
RE903	GAGGATCTGTTCCCGTTG	ΔLPKY ^{MBP}Mga2-LPKY-TMH; Q5 (r)
RE904	CGAATTAATGATGACTTACCATGTTATGAGGATCTGTTCCCG	*MGA2* K969C; QuikChange (f)

RE905	CGGGAACAGATCCTCATAACATGGTAAGTCATCATTAATTCG	*MGA2* K969C; QuikChange (r)
RE906	CGTTGTCTTGGGGTAAAGATGATTGTTTGAAAACCACAAATCAAGAC	*MGA2* K983C; QuikChange (f)
RE907	GTCTTGATTTGTGGTTTTCAAACAATCATCTTTACCCCAAGACAACG	*MGA2* K983C, QuikChange (r)
TP246	GCGAATTCCGAAATGATAAAATGTTAATATTTTTCTGTATACCCTTAACACTACTACTTTTG	*MGA2* W1042C; QuikChange (f)
TP267	CAAAAGTAGTAGTGTTAAGGGTATACAGAAAAATATTAACATTTTATCATTTCGGAATTCGC	*MGA2* W1042C; QuikChange (r)

Supplementary Table 4. Antibodies used for immunoblotting.

Antibody	Vendor	Catalogue #	Working dilution
Mouse anti-myc monoclonal (9E10)	Sigma-Aldrich	M4439	1:2,000
mouse anti-Pgk1 monoclonal (22C5)	Invitrogen	459250	1:20,000
mouse anti-MBP, monoclonal	NEB	E8032L	1:30,000
Goat anti-mouse-HRP	Dianova	115-035-146	1:20,000
Goat anti-mouse-IRDye 800CW	Li-COR	926-32210	1:20,000

The antibodies used in this study are listed along with the vendor, catalogue number, and the working solution are listed.

Supplementary Table 5. All-atom bilayers simulated in this study.

Bilayer	Construction	Lipids per leaflet	Waters per lipid	Ions	Simulation Time [ns]
DOPC	built with CHARMM-GUI membrane builder[15–17]	100	45	-	902 (915)
(1:1) DOPC:POPC		100	45	-	1008 (1009)
(5:1:4) DOPC:POPC:POPE		100	45	-	1018 (1018)
*trans*DOPC	Initial configuration taken from the end of CHARMM-GUI equilibration protocol[18]	100	45	-	973 (1007)

All-atom bilayers simulated in this study. Shown are (from left to right): bilayer composition, system construction details, number of lipids per leaflet, number of water molecules per lipid, number of ions in the system and the length of the last portion of the trajectory where the bilayer area is converged as determined by a previously reported algorithm[19]. The total simulation length is given in parenthesis.

Supplementary References

1. Covino, R. *et al.* A eukaryotic sensor for membrane lipid saturation. *Mol. Cell* **63**, 49–59 (2016).
2. Contreras, F.-X. *et al.* Molecular recognition of a single sphingolipid species by a protein's transmembrane domain. *Nature* **481**, 525–529 (2012).
3. Marrink, S. J., Risselada, H. J., Yefimov, S., Tieleman, D. P. & De Vries, A. H. The MARTINI force field: Coarse grained model for biomolecular simulations. *J. Phys. Chem. B* **111**, 7812–7824 (2007).
4. Monticelli, L. *et al.* The MARTINI coarse-grained force field: Extension to proteins. *J. Chem. Theory Comput.* **4**, 819–834 (2008).
5. Pettersen, E. F. *et al.* UCSF Chimera - A visualization system for exploratory research and analysis. *J. Comput. Chem.* **25**, 1605–1612 (2004).
6. Bussi, G., Donadio, D. & Parrinello, M. Canonical sampling through velocity rescaling. *J. Chem. Phys.* **126**, 014101 (2007).
7. Parrinello, M. & Rahman, A. Crystal structure and pair potentials: A molecular-dynamics study. *Phys. Rev. Lett.* **45**, 1196–1199 (1980).
8. Phillips, J. C. *et al.* Scalable molecular dynamics with NAMD. *Journal of Computational Chemistry* **26**, 1781–1802 (2005).
9. Klauda, J. B. *et al.* Update of the CHARMM All-Atom Additive Force Field for Lipids: Validation on Six Lipid Types. *J. Phys. Chem. B* **114**, 7830–7843 (2010).
10. Klauda, J. B., Monje, V., Kim, T. & Im, W. Improving the CHARMM force field for polyunsaturated fatty acid chains. *J. Phys. Chem. B* **116**, 9424–9431 (2012).
11. Doktorova, M., LeVine, M. V, Khelashvili, G. & Weinstein, H. A New Computational Method for Membrane Compressibility: Bilayer Mechanical Thickness Revisited. *Biophys. J.* **116**, 487–502 (2019).
12. Humphrey, W., Dalke, A. & Schulten, K. VMD: Visual molecular dynamics. *J. Mol. Graph.* **14**, 33–38 (1996).
13. Brachmann, C. B. *et al.* Designer deletion strains derived from Saccharomyces cerevisiae S288C: A useful set of strains and plasmids for PCR-mediated gene disruption and other applications. *Yeast* **14**, 115–132 (1998).
14. Giaever, G. *et al.* Functional profiling of the Saccharomyces cerevisiae genome. *Nature* **418**, 387–391 (2002).
15. Lee, J. *et al.* CHARMM-GUI Input Generator for NAMD, GROMACS, AMBER, OpenMM, and CHARMM/OpenMM Simulations Using the CHARMM36 Additive Force Field. *J. Chem. Theory Comput.* **12**, 405–413 (2016).
16. Wu, E. L. *et al.* CHARMM-GUI membrane builder toward realistic biological membrane simulations. *J. Comput. Chem.* **35**, 1997–2004 (2014).
17. Jo, S., Kim, T., Iyer, V. G. & Im, W. CHARMM-GUI: A web-based graphical user interface for CHARMM. *J. Comput. Chem.* **29**, 1859–1865 (2008).
18. Doktorova, M., Harries, D. & Khelashvili, G. Determination of bending rigidity and tilt modulus of lipid membranes from real-space fluctuation analysis of molecular dynamics simulations. *Phys. Chem. Chem. Phys.* **19**, 16806–16818 (2017).
19. Chodera, J. D. A Simple Method for Automated Equilibration Detection in Molecular Simulations. *J. Chem. Theory Comput.* **12**, 1799–1805 (2016).
